# Zero-heat-flux thermometry over the carotid artery in assessment of core temperature in craniotomy patients

**DOI:** 10.1007/s10877-023-00984-9

**Published:** 2023-03-06

**Authors:** Marja Silvasti-Lundell, Otto Makkonen, Riku Kivisaari, Teemu Luostarinen, Eero Pesonen, Marja-Tellervo Mäkinen

**Affiliations:** 1 Anaesthesiology and Intensive Care Medicine, University of Helsinki, Helsinki University Hospital, Helsinki, Finland; 2 Department of Neurosurgery, University of Helsinki and Helsinki University Hospital, Helsinki, Finland

**Keywords:** Body temperature, Zero-heat-flux temperature, Thermometry, Craniotomy

## Abstract

Zero-heat-flux core temperature measurements on the forehead (*ZHF-forehead*) show acceptable agreement with invasive core temperature measurements but are not always possible in general anesthesia. However, ZHF measurements over the carotid artery (*ZHF-neck*) have been shown reliable in cardiac surgery. We investigated these in non-cardiac surgery. In 99 craniotomy patients, we assessed agreement of *ZHF-forehead* and *ZHF-neck* (3M™ Bair Hugger™) with esophageal temperatures. We applied Bland-Altman analysis and calculated mean absolute differences (difference index) and proportion of differences within ± 0.5 °C (percentage index) during entire anesthesia and before and after esophageal temperature nadir. In Bland-Altman analysis [mean (limits of agreement)], agreement with esophageal temperature during entire anesthesia was 0.1 (−0.7 to +0.8) °C (*ZHF-neck*) and 0.0 (−0.8 to +0.8) °C (*ZHF-forehead*), and, after core temperature nadir, 0.1 (−0.5 to +0.7) °C and 0.1 (−0.6 to +0.8) °C, respectively. In difference index [median (interquartile range)], *ZHF-neck* and *ZHF-forehead* performed equally during entire anesthesia [*ZHF-neck*: 0.2 (0.1–0.3) °C vs *ZHF-forehead*: 0.2 (0.2–0.4) °C], and after core temperature nadir [0.2 (0.1–0.3) °C vs 0.2 (0.1–0.3) °C, respectively; all p > 0.017 after Bonferroni correction]. In percentage index [median (interquartile range)], both *ZHF-neck* [100 (92–100) %] and *ZHF-forehead* [100 (92–100) %] scored almost 100% after esophageal nadir. *ZHF-neck* measures core temperature as reliably as *ZHF-forehead* in non-cardiac surgery. *ZHF-neck* is an alternative to *ZHF-forehead* if the latter cannot be applied.

## Introduction

In spite of efforts to maintain normothermia, inadvertent perioperative hypothermia of patients is common [1, 2]. Even moderate hypothermia increases blood loss [3], cardiovascular events [4] and wound infections [5] and may prolong hospitalization [5] and increase mortality [6]. Conventional core body temperature measurements, e.g., pulmonary artery, esophagus, nasopharynx, or tympanum, are invasive [1]. A noninvasive zero-heat-flux (ZHF) technique for monitoring deep body temperature from the skin surface was originally introduced by Fox and Solman in 1970 [7] and thereafter further developed [8, 9]. Since 2014, an improved ZHF device (3M™ SpotOn™, later 3M™ Bair Hugger™) has been commercially available [10]. The system shows acceptable agreement with conventional core temperature measurements in cardiac [10] and non-cardiac [11–13] surgeries.

The patient sensor of the 3M™ Bair Hugger™ device is normally positioned on the lateral forehead, but in surgeries involving the head, requirements of sterility or the application of a surgical navigation device may hinder the use of ZHF probes here. Furthermore, if brain oxygenation is measured bilaterally with near infrared spectroscopy, there is no space for a ZHF probe on the forehead. Eshraghi and coworkers introduced positioning of ZHF sensors on the neck over the carotid artery in elective cardiac surgery. ZHF temperature measurements on the neck showed good accuracy (i.e., small bias), but slightly worse precision than anticipated, compared with pulmonary arterial temperatures [10].

Since rapid thermal perturbations are typical in cardiac surgery, Eshraghi and coworkers thought that the performance of ZHF temperature measurements over the carotid artery might be better in non-cardiac surgery [10]. In the present prospective observational study, we evaluated the agreement of ZHF temperature measurements on the skin over the carotid artery with esophageal temperatures in patients undergoing elective craniotomy.

## Materials and methods

### Patients

The Ethics Committee of Hospital District of Helsinki and Uusimaa, Helsinki, Finland provided ethical approval for this study (Ethics Committee for Surgery of the Hospital District of Helsinki and Uusimaa, Helsinki, Finland; diary number HUS/521/2019). Before surgery, all patients gave written informed consent to participate in this study. We aimed to recruit 100 patients aged 18–90 years and scheduled for elective craniotomy. There were no exclusion criteria.

### Anesthesia

Patients received diazepam as premedication. We induced anesthesia with thiopental sodium or propofol, fentanyl and rocuronium. We maintained anesthesia either with intravenous propofol infusion, with 1 MAC of isoflurane or sevoflurane in combination with 50% nitrous oxide in oxygen, or with a combination of propofol and isoflurane/sevoflurane. We supplemented anesthesia with remifentanil infusion and rocuronium boluses. In mechanical ventilation (Datex-Ohmeda Aisys, Madison, WI, USA), the target of arterial pCO_2_ was 4.0–4.5 kPa. We displayed respiratory and anesthetic gas concentrations, spirometry, oxygen saturation, electrocardiogram and invasive arterial blood pressure on Datex-Ohmeda S/5 Anesthesia Monitor (Helsinki, Finland).

### Temperature measurement

Before anesthesia induction, we placed a ZHF temperature-monitoring Bair Hugger sensor (3M, St Paul, MN, USA) in two positions contralateral to the site of the operation: 1) on the skin of the forehead at the mid-orbital line above the eyebrow (*ZHF-forehead*), and 2) on the skin over the maximal carotid arterial pulse under the jaw between the sternocleidomastoid muscles (*ZHF-neck*). At the time of tracheal intubation, we inserted an esophageal temperature probe (CareFusion Temperature Probe, 12 Fr, CareFusion Finland 320 Ltd, Helsinki, Finland) through the nose at a depth estimated from the patient’s height, as previously described [14, 15]. After anesthesia induction, according to clinical routine, we inserted a urinary bladder catheter (Mon-a-Therm Foley Catheter with Temperature Sensor 400TM, Covidien, Mansfield, MA, USA).

We recorded body temperatures continuously in an automated anesthesia system (Caresuite Anesthesia Manager 8.0, Picis Inc., Wakefield, MA, USA). From the hospital database, we retrieved temperature measurements at five-minute intervals, considered long enough to detect a relevant change in body temperature between consecutive time points. We recorded temperature measurements starting after each temperature sensor had reached equilibrium. We continued measurements until removal of the esophageal temperature probe at the end of the operation.

### Temperature management

We warmed patients actively during surgery using a forced-air warming blanket (Bair Hugger Therapy, 3M, St Paul, MN, USA), an over-body conductive blanket (Hot Dog Augustine Temperature Management, Eden Prairie, MN, USA), or a warming mattress (Hot Dog Augustine Temperature Management, Eden Prairie, MN, USA). Additionally, we applied warmed cotton blankets or space blankets if needed. We maintained operating room temperatures at 19–22 °C.

### Measurement of the carotid arterial depth

We placed one ZHF probe over the site of the maximal carotid arterial pulse. Since the Bair Hugger ZHF sensor reaches a depth of 1–2 cm [10, 16], the same anesthesiologist (MSL) measured the depth of the carotid artery for all patients, using ultrasound as follows: A linear ultrasound probe (Logiq e, GE Healthcare, Jiangsu, P. R. China) was placed tangentially on the skin, positioning the carotid artery in the middle of the ultrasound image. To avoid any compression of subcutaneous tissue, the pressure of the ultrasound probe was gradually reduced but with the carotid artery still visualized. The ultrasound image was fixed and the perpendicular distances of the upper and lower walls of the carotid artery from the skin surface measured. Measurements were repeated three to seven times and the means calculated.

### Statistical analysis

A power analysis was not undertaken for this observational study. We aimed at recruiting 100 patients, expecting this number of patients to be allocated to the dedicated study anesthesiologist (MSL) within one year. The esophageal temperature was used as the reference core temperature measurement [17]. The primary objective was to assess the agreement of *ZHF-neck* and *ZHF-forehead* temperatures with esophageal temperatures. Two agreement indices were calculated for each patient: as the “percentage agreement index”, the percentage of measurement differences within the range of ± 0.5°C was counted; as the “difference agreement index”, the mean of absolute measurement differences was calculated. We recognized two confounding factors for *ZHF-neck*. First, rotation of the neck in a lateral surgical position may result in malpositioning of the ZHF-probe relative to the carotid artery. Second, the carotid artery may be deeper than 2 cm in the subcutaneous tissue of obese patients. In addition to the agreement indices, the Bland-Altman random-effects approach for data of repeated measures was assessed for both ZHF methods, relative to the esophageal temperature as reference [18]. General anesthesia has major impacts on thermoregulation [19, 20, 21]. The period of general anesthesia was divided into “early phase” and “late phase”, according to the nadir of the esophageal temperature. Statistical analyses were conducted separately for the early phase, the late phase, and the entire study period.

We used MedCalc® statistical software (MedCalc Software, Ostend, Belgium) for statistical analyses. The percentage and difference agreement indices were not normally distributed, even after logarithmic transformation. BMIs and carotid arterial depths were normally distributed. Wilcoxon signed-rank test was used to test differences in the agreement indices between the *ZHF-neck* and *ZHF-forehead* methods, and Mann-Whitney U-tests to test differences in the agreement indices between the patients operated on in supine and lateral positions. Bivariate correlations were tested between BMI and carotid arterial depth with Pearson’s correlation test, and correlations involving the agreement indices with Spearman’s rho test. Patient and operative data were expressed as mean ± standard deviation, the Bland-Altman bias as mean and limits of agreement, Lin’s concordance correlation coefficient with 95% confidence intervals (CI), and the agreement indices as median and interquartile range (IQR). P-values < 0.05 were considered statistically significant, except < 0.17 after Bonferroni correction of the parallel analyses of the three different anesthesia periods (entire period, early phase, late phase). The STROBE guidelines were applied.

## Results

**Fig. 1:**
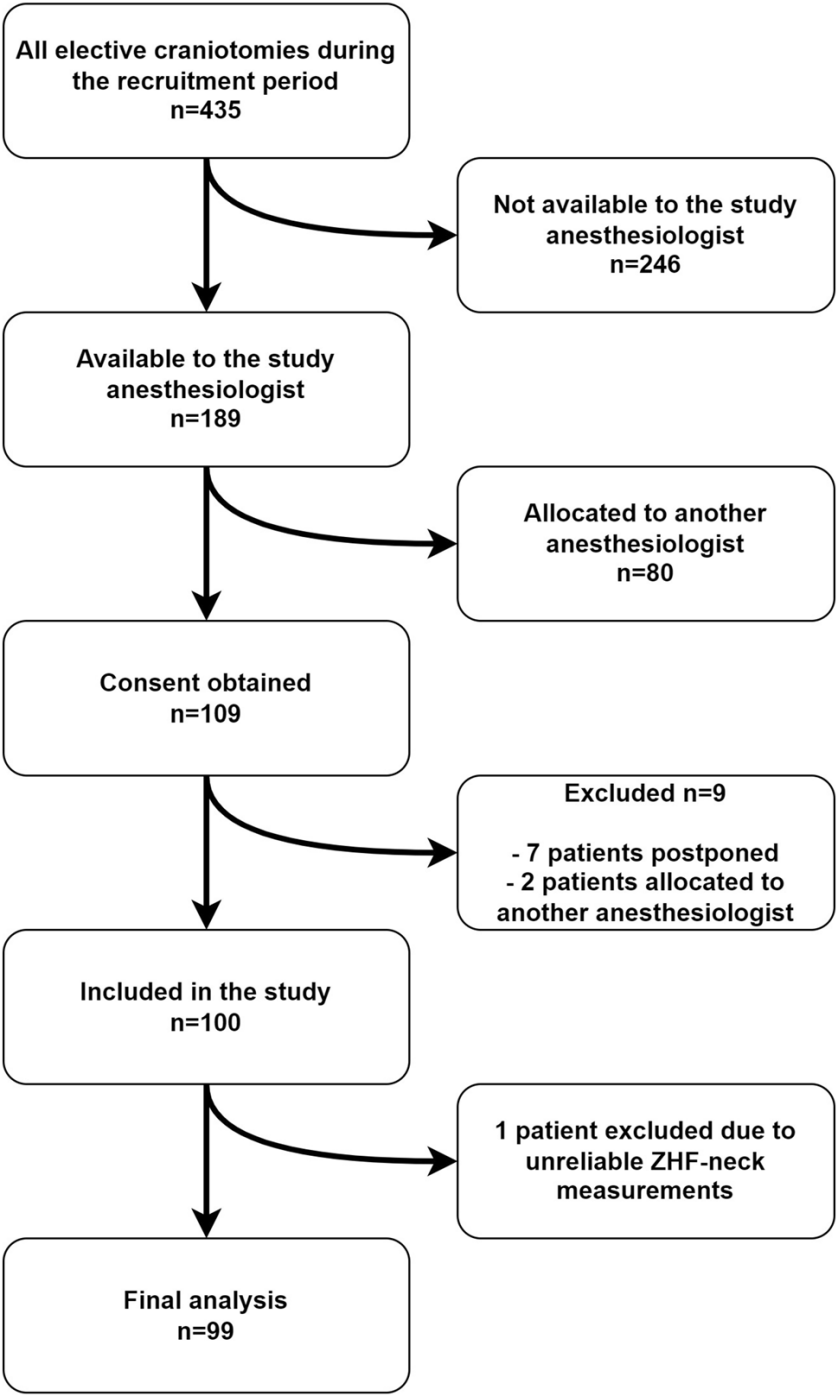
Flow chart

During patient recruitment, 425 elective craniotomies were performed in the study location. Of these, 189 were performed when the dedicated study anesthesiologist (MSL) was on duty. Of these patients, 109 were allocated to her care, of whom 100 entered the study. We excluded one patient from the final analysis for a technical reason. The patient flow chart is shown in Fig. 1. Seventy patients were operated on in a supine position and 28 in a lateral one. In addition, one 64-year-old female (BMI 26 kg/m², surgery duration 5.3 hours, anesthesia duration 6.5 hours) was operated on in a prone position. The mean depth of the anterior wall of the carotid artery was 1.3 ± 0.4 cm, and the mean BMI was 27.4 ± 5.6 kg/m^2^. The patient and operational data are presented in Table 1.

**Table 1:** Patient and operative data

	All patients^a^	Supine position	Lateral position
Number of patients	99	70	28
Age (years)	58 ± 13	57 ± 13	59 ± 14
Height (cm)	170 ± 8	170 ± 8	171 ± 8
Weight (kg)	79 ± 18	81 ± 19	76 ± 15
BMI (kg/m²)	27 ± 6	28 ± 6	26 ± 5
Sex (male/female)	37 / 62	24 / 46	13 / 15
Duration of anesthesia (min)	238 ± 68	222 ± 62	272 ± 65
Duration of surgery (min)	156 ± 65	144 ± 61	178 ± 65
Anesthetic (propofol/sevoflurane/isoflurane)	98/2/1	69/2/1^b^	28/0/0

Values presented as mean ± standard deviation

^a^One patient was in a prone position and included only in the overall analysis of the patients

^b^Two patients were anesthetized with a combination of propofol and sevoflurane

**Fig. 2:**
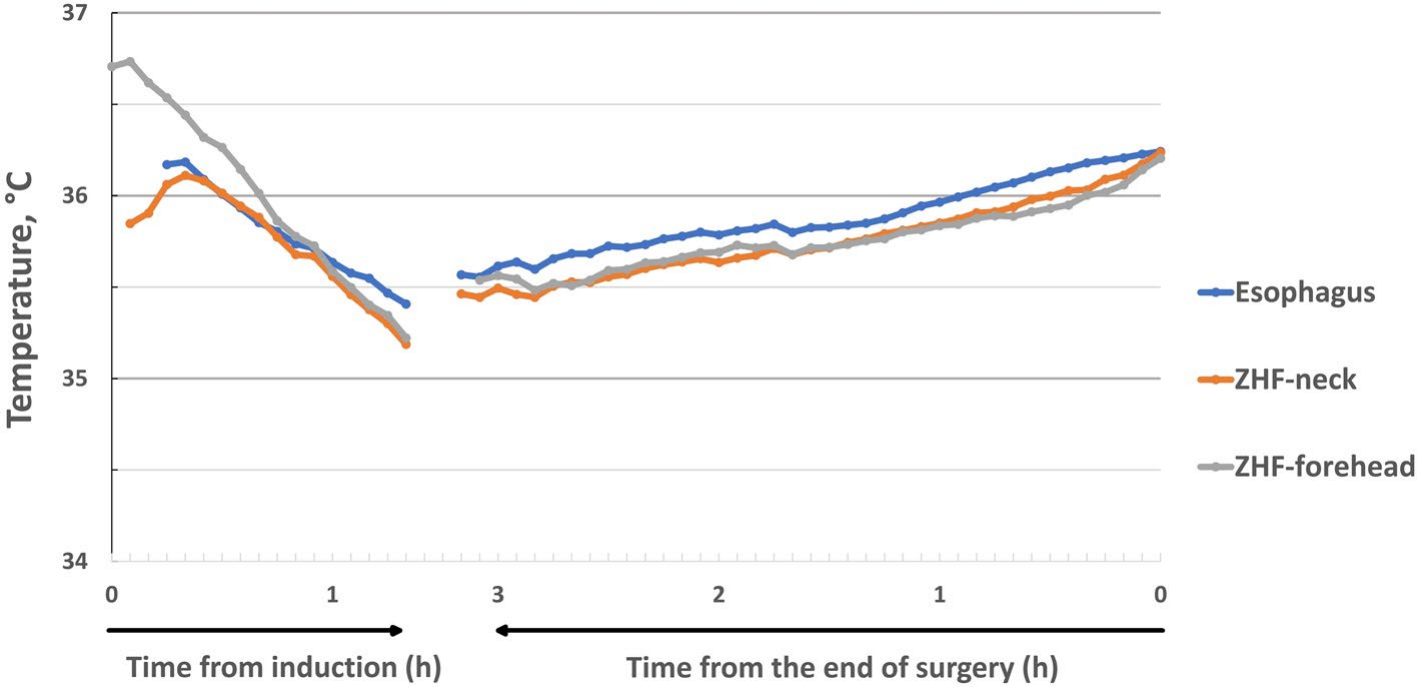
Progression of the temperature measurement methods as a function of time expressed as means. The length of an hour marked on the time axis.

Progression of the different core temperature measurements is shown in Fig. 2. In the early phase, different declining patterns of the core temperatures were observed. The *ZHF-forehead* temperature caught up with the *ZHF-neck* and esophageal temperatures by the one-hour mark. In the late phase, all core temperature methods paralleled each other within a range of less than 0.5 °C (Fig. 2).

**Fig. 3:**
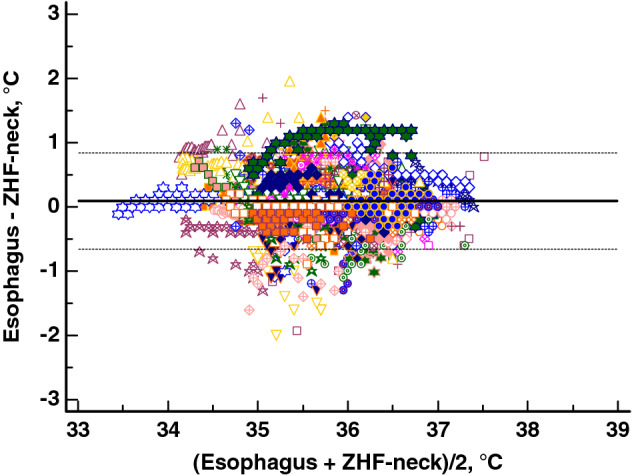
Bland-Altman plot for data from esophageal vs *ZHF-neck* temperatures during the entire period, showing 95% limits of agreement (dotted line). Each patient is depicted with a different symbol.

**Fig. 4:**
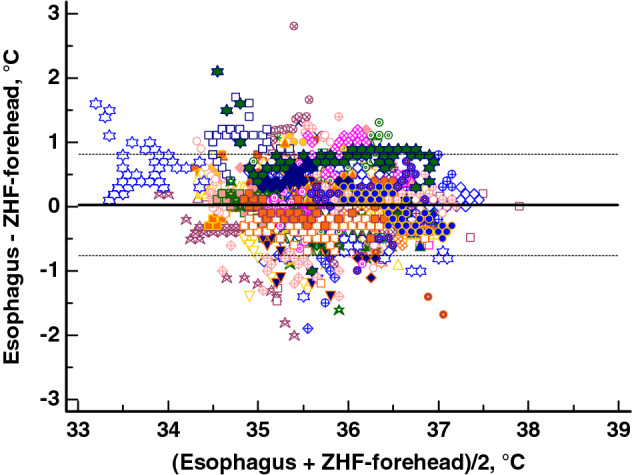
Bland-Altman plot for data from esophageal vs *ZHF-forehead* temperatures during the entire period, showing 95% limits of agreement (dotted line). Each patient is depicted with a different symbol.

Altogether, we detected 4289 esophagus/*ZHF-neck* and 4091 esophagus/*ZHF-forehead* pairs of temperature measurements at intervals of five minutes. We evaluated agreements with the Bland-Altman method for the whole operation period and separately for the early and late phases of surgery. The resulting mean differences are listed in Table 2. The Bland-Altman plot with the esophageal temperature against *ZHF-neck* temperature is shown in Fig. 3 and against *ZHF-forehead* temperature in Fig. 4. Esophageal and ZHF-temperatures were also evaluated with Lin’s concordance correlation coefficient. The results are listed in Table 2.

**Table 2:** Bland-Altman limits of agreement, agreement indices and Lin’s correlations

		Entire period	Early phase	Late phase
Bland-Altman: Bias and limits of agreement	Forehead	0.0 (− 0.8 to +0.8)	− 0.1 (− 1.0 to +0.7)	0.1 (− 0.6 to +0.8)
	Neck	0.1 (− 0.7 to +0.8)	0.0 (− 1.0 to +1.1)	0.1 (− 0.5 to +0.7)
Lin’s concordance correlation coefficient (95% CI)	Forehead	0.85 (0.84–0.86)	0.81 (0.79–0.83)	0.87 (0.86–0.88)
	Neck	0.85 (0.84–0.86)	0.74 (0.72–0.77)	0.88 (0.87–0.89)
Difference agreement index: median (IQR)	Forehead	0.2 (0.2–0.4)	0.3 (0.2–0.4)	0.2 (0.1–0.3)
	Neck	0.2 (0.1–0.3)	0.3 (0.2–0.5)	0.2 (0.1–0.3)
Percentage agreement index (%): median (IQR)	Forehead	93 (83–100)	92 (78–100)	100 (92–100)
	Neck	96 (84–100)	90 (64–100)	100 (92–100)

*IQR* Interquartile range; *CI* confidence interval

Neither the mean difference nor the percentage agreement indices differed statistically significantly between the ZHF methods in any of the time periods (Table 2). *ZHF-neck* temperature did not differ between the supine and lateral positions for either index in any period (Table 3).

**Table 3:** Agreement indices operated on in supine vs lateral position *IQR* Interquartile range

			Entire period	Early phase	Late phase
Supine	Difference agreement index: median (IQR)	Forehead	0.2 (0.2–0.4)	0.3 (0.2–0.4)	0.2 (0.1–0.3)
		Neck	0.2 (0.1–0.3)	0.3 (0.2–0.5)	0.2 (0.1–0.3)
	Percentage agreement index (%): median (IQR)	Forehead	96 (80–100)	93 (80–100)	100 (90–100)
		Neck	96 (85–100)	91 (63–100)	100 (93–100)
Lateral	Difference agreement index: median (IQR)	Forehead	0.2 (0.2–0.3)	0.3 (0.2–0.4)	0.2 (0.1–0.3)
		Neck	0.2 (0.1–0.4)	0.3 (0.2–0.5)	0.2 (0.1–0.3)
	Percentage agreement index (%): median (IQR)	Forehead	93 (86–98)	89 (72–100)	100 (92–100)
		Neck	95 (85–100)	85 (67–100)	100 (91–100)

BMI and carotid arterial front wall depth correlated with each other (R = 0.39, p < 0.01). Neither correlated significantly with the *ZHF-neck* mean difference agreement index (carotid wall depth: rho = 0.05, p = 0.64; BMI: rho = 0.07, p = 0.49) nor with the percentage index (carotid wall depth: rho = − 0.01, p = 0.88; BMI: rho = − 0.11, p = 0.27).

## Discussion

ZHF temperature measurements over the carotid arterial pulse (*ZHF-neck*) showed comparable performance with the conventional application of ZHF on the forehead (*ZHF-forehead*). Both measurement locations reached good measurement accuracy compared with esophageal temperatures. As in previous studies [10, 22], however, measurement precision in both locations was slightly outside the preferred range of ± 0.5 °C. Neither lateral surgical position nor obesity impaired the performance of *ZHF-neck* thermometry.

As the carotid arteries are close to the central arterial circulation, we hypothesized that the subcutaneous tissue around these arteries would represent the core temperature compartment. In the present study, the measurement accuracy of *ZHF-neck* temperatures, compared with esophageal temperatures as reference, was good, with a mean difference (bias) of 0.1 °C in Bland-Altman analysis. The measurement precision with 95% limits of agreement of ± 0.8°C did not reach the commonly used criterion of good precision of ± 0.5 °C [23]. The agreement between *ZHF-forehead* and esophageal temperatures of 0.0 (− 0.8 to +0.8) °C was of the same magnitude as we have previously reported between *ZHF-forehead* and nasopharyngeal temperatures [13].

The Bair Hugger ZHF sensor used in this study is reported to reach depths of 1–2 cm [10, 16]. In our patients, the mean depth of the anterior wall of the carotid artery was 1.3 cm, ranging from 0.4 to 2.2 cm. As expected, the carotid arterial depth correlated with BMI. Neither the depth of the carotid artery nor BMI correlated with either of the agreement indices between *ZHF-neck* and esophageal temperatures. These findings do not necessarily indicate that the *ZHF-neck* probe reaches the carotid artery. Instead, they suggest that the depth of the carotid artery or BMI do not confound *ZHF-neck* temperature measurements. Furthermore, in Bland-Altman analysis between the *ZHF-neck* and esophageal temperatures, there was no clinically meaningful correlation between the methods’ temperature mean (i.e., actual body temperature) and the temperature difference (i.e., measurement bias). This suggests that the *ZHF-neck* temperature was reliable at least within the body temperature range of the present study [18]. Finally, the performance of *ZHF-neck* temperature measurements did not differ between the patients operated on in supine or lateral positions.

To our knowledge, there are only two previous studies applying ZHF to the neck. In a study of ten patients undergoing laparoscopic surgery, ZHF on the neck showed a mean difference of 0.05 °C in Bland-Altman analysis (95% limits of agreement of ± 0.35 °C) compared with esophageal temperatures [24]. In cardiac surgery, the average intraoperative temperature difference between *ZHF-neck* and pulmonary arterial temperatures was 0.15 °C (95% limits of agreement of ± 0.84 °C). Of all measurements, 81% showed differences of ≤ 0.5 °C [10]. Obesity did not impair temperature measurements. *ZHF-neck* temperatures were comparable to ZHF temperatures measured on the forehead [10]. All these findings are in agreement with our results.

This study is the first to analyze ZHF temperature measurements separately for the early and late phases of anesthesia. In awake individuals, core temperature values vary between different locations [25]. Probably reflecting normal physiology and corroborating our previous findings [13], *ZHF-forehead* was more than 0.5 °C higher than esophageal temperatures at anesthesia induction. General anesthesia has a major impact on thermoregulation [19]: during the first 1–2 hours of anesthesia, redistribution of body heat occurs from the core towards the body surface and temperature gradients between different anatomical locations decline [20, 21]. In our patients, at anesthesia induction, the discordant core temperature readings probably represent actual temperatures of the different anatomical locations. During the late phase, i.e., after initial redistribution of body temperature, both *ZHF-neck* and *ZHF-forehead* paralleled closely esophageal temperatures. The limits of agreement between *ZHF-neck* and esophageal temperature during the late phase of anesthesia was as good as + 0.1 ± 0.6 °C and the percentage indices scored close to 100%. These are important pieces of information: these results suggest that both ZHF methods reflect esophageal temperature closely only when its nadir has been reached. This could partly explain why measurement precision between *ZHF-forehead* and invasive control temperature measurements did not reach the preferred range of ± 0.5 °C when measurements during the entire general anesthesia duration were processed together as uniform data [22].

Our study has limitations. First, we could not measure pulmonary arterial temperature, the gold standard for core body temperature. Instead, we used esophageal temperature as the reference [17]. We paid attention to the optimal depth of the probe placement [14, 15]. Second, we did not conduct a power analysis but estimated that the dedicated study anesthesiologist could recruit one hundred patients in a year. Third, the fact that this anesthesiologist conducted all measurements reduces the generalizability of the results. One strength of this study is that automated continuous temperature recording enabled investigation of the interrelationship of different temperature measurement methods in different phases of anesthesia. Second, an interval as long as five minutes between two consecutive recordings ensured that, instead of simple repetition, we could detect true body temperature changes. Recording interval is an important factor when considering the credence of the limits of agreements in Bland-Altman analyses [18].

In conclusion, non-invasive ZHF temperature measurements over the carotid arterial pulse reliably measure core body temperatures in non-cardiac surgery. Carotid arterial ZHF offers an alternative in clinical situations where ZHF measurements on the forehead cannot be made. Importantly, major clinical factors like obesity, surgical position and temperature range do not confound ZHF temperature measurements over the carotid artery.
